# New insights into subcomplex assembly and modifications of centrosomal proteins

**DOI:** 10.1186/1747-1028-7-17

**Published:** 2012-07-16

**Authors:** Karin Habermann, Bodo MH Lange

**Affiliations:** 1 Department of Vertebrate Genomics, Max Planck Institute for Molecular Genetics, Berlin, Germany

**Keywords:** Centrosome, γ-TuRC, Augmin/HAUS complex

## Abstract

This review provides a brief overview of the recent work on centrosome proteomics, protein complex identification and functional characterization with an emphasis on the literature of the last three years. Proteomics, genetic screens and comparative genomics studies in different model organisms have almost exhaustively identified the molecular components of the centrosome. However, much knowledge is still missing on the protein-protein interactions, protein modifications and molecular changes the centrosome undergoes throughout the cell cycle and development. The dynamic nature of this large multi-protein complex is reflected in the variety of annotated subcellular locations and biological processes of its proposed components. Some centrosomal proteins and complexes have been studied intensively in different organisms and provided detailed insight into centrosome functions. For example, the molecular, structural and functional characterization of the γ-Tubulin ring complex (γ-TuRC) and the the discovery of the Augmin/HAUS complex has advanced our understanding of microtubule (MT) capture, nucleation and organization. Surprising findings revealed new functions and localizations of proteins that were previously regarded as bona fide centriolar or centrosome components, e.g. at the kinetochore or in the nuclear pore complex regulating MT plus end capture or mRNA processing. Many centrosome components undergo posttranslational modifications such as phosphorylation, SUMOylation and ubiquitylation that are critical in modulating centrosome function and biology. A wealth of information has recently become available driven by new developments in technologies such as mass spectrometry, light and electron microscopy providing more detailed molecular and structural definition of the centrosome and particular roles of proteins throughout the cell cycle and development.

## Centrosome proteome

**Figure 1:**
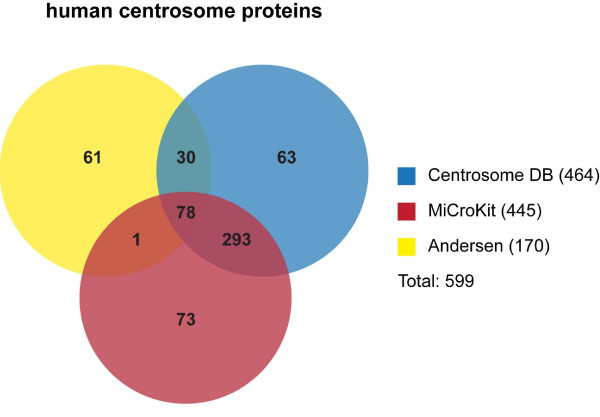
**Overlap of human centrosome proteins in 3 different datasets. **Venn diagram illustrating the overlap of human centrosome proteins annotated in Centrosome:db [21], MiCroKit [22] and centrosome proteins identified in a proteomics/antibody screen of the Andersen lab [7].

Recent work has provided a wealth of new molecular and functional information on the centrosome in a wide variety of organisms. Several large-scale RNA interference (RNAi) screens [1-4], proteomics [5-16] and comparative genomics studies [17-20] have identified the molecular components of the centrosome and related structures such as the spindle pole body, cilia and flagella. However, the exact number of centrosomal proteins is difficult to determine since especially the mass spectrometry (MS)-based studies suffer from the fact that true components can be lost and contaminants are included when isolating organelles such as the centrosome. Furthermore, many proteins only transiently associate with centrosomes and less abundant proteins may escape identification due to the limited sensitivity of MS techniques. Nevertheless the list of identified centrosome proteins continuously grows. *Centrosome:db*, a database devoted to the human centrosome proteome, currently contains a total of 464 genes encoding proteins that stably or transiently localize to the centrosome [21]. Genes were added on the basis of different kinds of evidence, including high-throughput proteomics datasets and Gene Ontology (GO) annotation in Ensembl and the Human Protein Reference Database (HPRD). A second collection of centrosome proteins can be found in the MiCroKit database [22], which extracts proteins from scientific literature that have been shown to localize to the centrosome, midbody and/or kinetochore by fluorescence microscopy in 7 different model organisms. MiCroKit currently lists 445 human centrosome proteins, of which 371 are also present in Centrosome:db. Andersen and colleagues contributed a large fraction of centrosomal proteins (108) to these databases by examining the human interphase centrosome by MS. Their initial analysis resulted in the identification of roughly 500 proteins. In order to discriminate between contaminants and true centrosome components, they used protein correlation profiling (PCP) and validated a subset of novel candidates via GFP tagging and localization studies [5]. Recently an advanced follow-up study, which combined protein identification by PCP-SILAC (stable isotope labeling by amino acids)-MS and protein localization by BAC TransgeneOmics and antibody screening, identified 170 human centrosome proteins [7]. These include all previously reported ones and 61 new candidates that have not yet been incorporated into Centrosome:db or MiCroKit. Importantly this study also provides information on novel asymmetrically localizing centrosome components, stoichiometry of subcomplexes and turnover rates of centrosome proteins. When combining the data from Centrosome:db, MiCroKit and the latest Andersen study, the number of human centrosome components approaches 600 (Figure 1), twice as many as suggested earlier [23]. However, the comprehensive knowledge about the molecular inventory of the centrosome comprises only a fraction of the information we need to understand this cell organelle. In the coming years it will be increasingly important to define the protein-protein interactions, protein modifications and molecular changes the centrosome undergoes throughout the cell cycle and development.

## Centrosome functions

**Figure 2:**
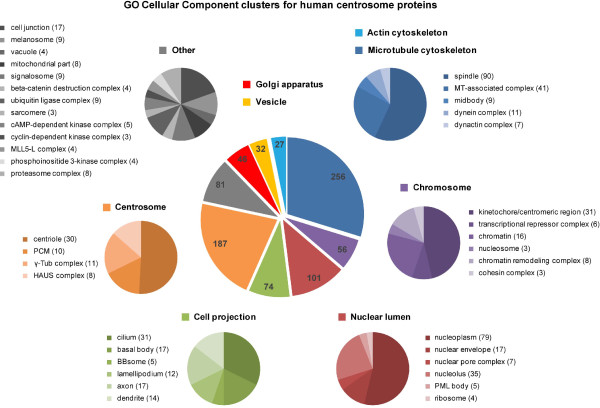
**GO Cellular Component clusters for human centrosome proteins. **The subcellular localizations of the 599 centrosome candidate proteins were extracted with the Database for Annotation, Visualization and Integrated Discovery (DAVID), version 6.7 [48] using the Gene Ontology (GO) term ‘’Cellular Component”. The distribution of proteins over 9 main clusters (“actin cytoskeleton”, light blue; “MT cytoskeleton”, blue; “chromosome”, purple; “nuclear lumen”, red; “cell projection”, green; “centrosome”, orange; “other”, grey; “golgi apparatus”, deep red; “vesicle”, yellow) is illustrated as pie chart and the number of proteins annotated for each GO term is indicated. Subclusters within 7 of the 9 main clusters are shown in the respective colors and the number of proteins annotated for each GO term is indicated in parenthesis. Note that pie charts are simplified representations that do not reflect 100%. Proteins can have multiple annotations or no annotation. This is not illustrated in the charts.

**Figure 3:**
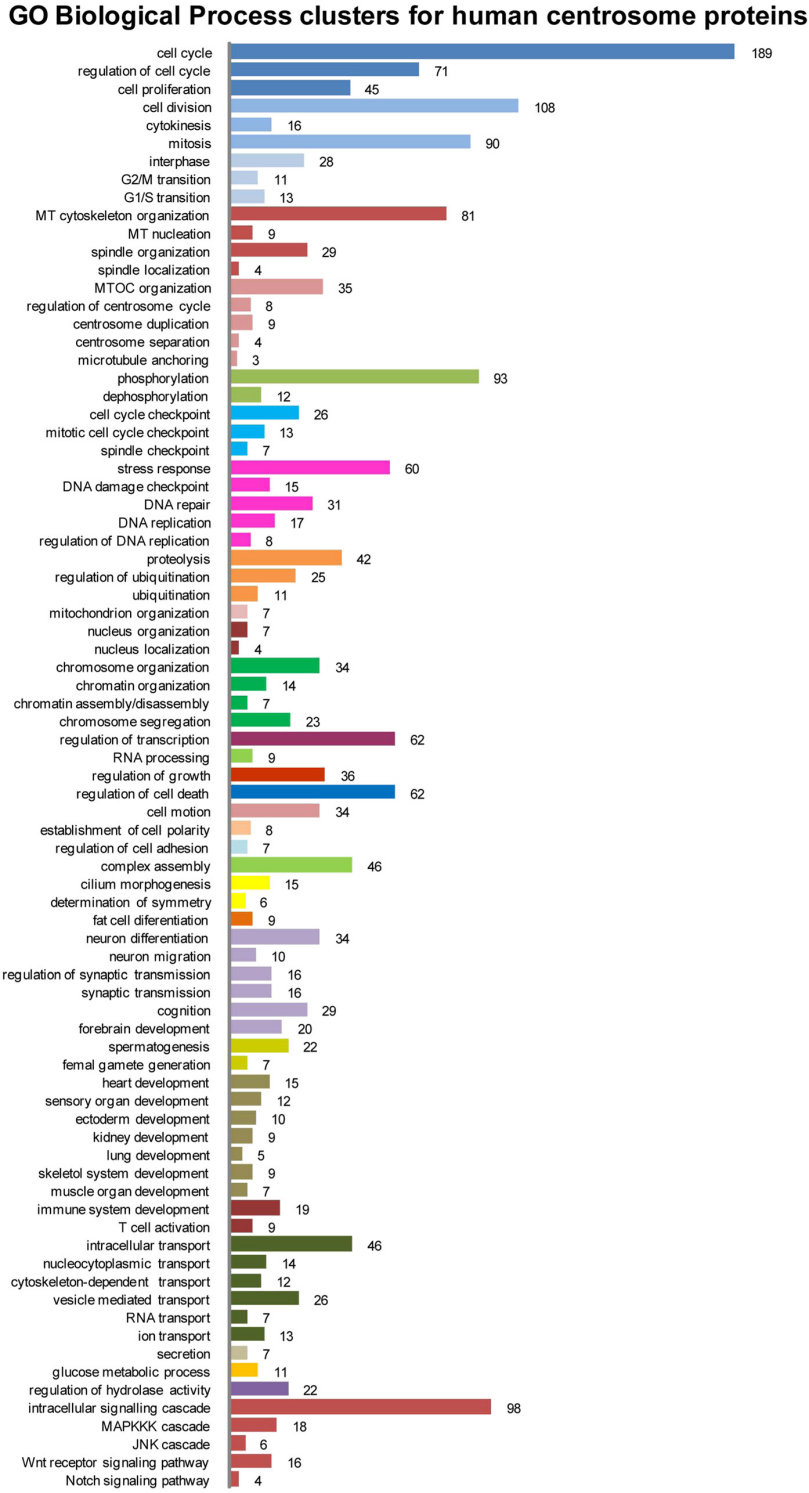
**GO Biological Process clusters for human centrosome proteins. **Functions of the 599 centrosome candidate proteins were annotated with the DAVID tool using the GO term ‘’Biological process”. The graph illustrates the number of proteins annotated with 79 different GO terms. Related terms were grouped and are shown in the same color, e.g. cell cycle checkpoint, mitotic cell cycle checkpoint and spindle checkpoint in light blue.

The centrosome is a multifunctional complex, which is also reflected in the variety of annotated subcellular locations and biological processes of its proposed components (Figure 2 and 3). The most prominent role of the centrosome is the nucleation and organization of MTs and hence the coordination of all MT-dependent functions. In interphase cells, this includes the regulation of cell motility, adhesion and polarity, the maintenance of cell shape as well as intracellular transport and positioning of organelles. In proliferating cells, the centrosome facilitates the assembly of the bipolar mitotic spindle, which is required for efficient and correct segregation of duplicated chromosomes [23-25]. While centrosomes may be dispensable for spindle assembly in some cell types [26,27], centrioles (the cylinder shaped structures embedded in the pericentriolar matrix) are absolutely essential for the organization of cilia and flagella. Nonmotile primary cilia, which are present in most vertebrate cells, play a role in chemical sensation and modulate several signaling pathways that are essential during development [28]. Ciliary defects are linked to a variety of diseases, such as abnormalities in left-right asymmetry (situs inversus) [29], polycystic kidney and liver disease and other ciliopathic genetic disorders like the Bardet-Biedl syndrome, which is caused by mutations in different basal body genes [30]. The centrosome is furthermore involved in the orchestration of various cell cycle events, such as entry into mitosis, anaphase onset, cytokinesis and the G1 to S transition [31]. Several findings suggest that centrosomes are implicated in signaling pathways related to stress response: When cells are exposed to elevated temperatures, centrosomes disperse and multipolar spindles are formed [32]. Similarly, centrosome fragmentation and inactivation has been shown to occur in response to impaired DNA integrity in both DNA damage checkpoint defective vertebrate cells and *Drosophila* embryos [33-36]. Moreover, heat shock proteins, e.g. Hsp90 [37] and components of the DNA damage checkpoint (Chk2, Chk1) are concentrated at the centrosome [34,38]. However, the molecular mechanisms which lead to disruption and inactivation of centrosomes in response to environmental perturbations remain to be fully understood. Lastly, evidence for a direct link between centrosome aberrations and tumorigenesis was found by several independent studies leading to our current understanding that centrosome amplification is sufficient to promote tumor formation by inducing relatively low levels of chromosome missegregation [39-45]. Reviews that highlight the wide spectrum of centrosome protein function are for example [46,47].

Clearly the centrosome is involved in a multitude of cellular processes, yet the underlying molecular mechanisms by which it coordinates these processes largely remain unknown. Therefore the present challenge is to define the functional and physical connectivity within the centrosomal proteome and to investigate the association of centrosomal subcomplexes to the various cellular signaling pathways. In the next sections, recent discoveries in the field of intrinsic centrosome protein-protein interactions redefining well-known and identifying novel centrosome-associated complexes will be elaborated.

## Centrosome complexes: γ-Tubulin ring complex and Augmin/HAUS

The best studied among the centrosomal complexes is the highly conserved γ-TuRC, which is responsible for the nucleation of MTs from centrosomes and also from chromosomes and, as recently reported, from within the mitotic spindle [49]. In humans, this complex comprises at least 7 subunits, γ-Tubulin (also known as GCP1) and the γ-Tubulin complex proteins (GCP2-6 and NEDD1 (also known as GCP-WD)) [50-54]. 2 copies of γ-Tubulin together with GCP2 and GCP3 form a tetramer termed γ-Tubulin small complex (γ-TuSC) [55,56]. Several γ-TuSCs assemble into a higher order ring structure, in which GCPs 4–6 form a stabilizing cap on one side of the ring [57]. NEDD1, which was later revealed as a homologue of the Drosophila γ-TuRC subunit Dgp71WD by sequence comparison, is now also defined as a core component of the human γ-TuRC although it is dispensable for its assembly and does not share sequence similarity with the other GCPs. However, it is required for both centrosomal- and chromatin-mediated MT nucleation by targeting γ-TuRC to these sites [54]. Spindle localization of the γ-TuRC was furthermore shown to depend on mitotic phosphorylation of NEDD1 [54]. The human microcephaly protein CDK5RAP2/CEP215 also interacts with the γ-TuRC and functions in attaching γ-Tubulin to the centrosome via its γ-Tubulin complex binding domain [58]. Moreover this domain was shown to stimulate the MT nucleation capacity of the γ-TuRC in vitro while it is not required for complex assembly [59]. Recently new interactors of the γ-TuRC were identified in a high-throughput analysis of human protein complexes by the MitoCheck consortium [60]. The authors used BAC TransgeneOmics, localization studies and tandem affinity purification of more than 200 bait proteins to investigate protein complexes from human tissue culture cells arrested in mitosis. In this study NME7, which belongs to the nucleoside diphosphate kinase family, Galectin-3-binding protein LGALS3BP, MOZART1 as well as the two closely related proteins MOZART2A and B were found to associate with subunits of the γ-TuRC [60]. With the exception of LGALS3BP all proteins were shown to localize to the centrosome and mitotic spindles. Subsequent functional analysis of MOZART1 revealed that this protein is involved in mitotic spindle assembly by recruiting γ-TuRC to centrosomes [60]. MOZART2 has meanwhile been proposed to be a novel core subunit of the γ-TuRC, as it cofractionates with γ-Tubulin and GCP5 at a size expected for the γ-TuRC in sedimentation assays [61]. Furthermore, it localizes to centrosomes throughout the cell cycle in a NEDD1-dependent fashion and to the spindle in mitosis, as do the known γ-TuRC components. Similar to NEDD1, CDK5RAP2 and MOZART1, MOZART2 is not sequence-related to GCPs and has no structural role in the γ-TuRC. However, it was demonstrated to be involved in centrosomal recruitment of γ-Tubulin, NEDD1 and CDK5RAP2 and affecting the nucleation activity of centrosomes specifically in interphase of the cell cycle, a function that is distinct from other γ-TuRC subunits [61]. The authors of this study suggest to rename MOZART2 to GCP8 according to the nomenclature for core components of the γ-TuRC, counting NEDD1 as GCP7. Whether MOZART1 also qualifies for a core γ-TuRC subunit remains to be investigated. In addition to identifying a novel core subunit of the γ-TuRC, this study also revealed cell-cycle dependent and low affinity interaction partners. NME7, LGALS3BP, components of the CCT chaperonin complex, RUVBL1 and RUVBL2 were present in the purified γ-TuRCs in interphase and mitosis but less abundant as the core subunits. The mitotic kinase Plk1 and 7 of the 8 Augmin/HAUS complex subunits were found to preferentially associate with γ-TuRCs in mitosis.

**Figure 4:**
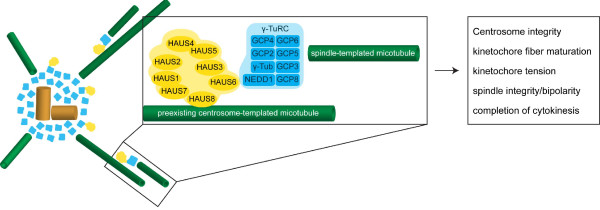
**Model for HAUS- and γ-TuRC-mediated MT generation within the mitotic spindle (adapted from Uehara et al. **[63]**). **The 8-subunit HAUS complex, which resides at the centrosome in interphase, recruits γ-TuRCs from centrosomes to spindle MTs in mitosis, where γ-TuRC nucleates additional MTs. HAUS8/HICE1 directly binds to MTs and to HAUS6/FAM29A, which in turn binds the γ-TuRC via NEDD1. HAUS complex and γ-TuRC are shown in yellow and blue, respectively. The components of both complexes are indicated in the magnified view. Spindle-templated MT amplification mediated through interaction of the HAUS complex and the γ-TuRC was demonstrated to be critical for maintenance of centrosome and spindle integrity, for the generation of tension between sister kinetochores and for the completion of cytokinesis.

Components of the Augmin complex were originally discovered in a large-scale RNAi screen in search of genes involved in mitotic spindle assembly in Drosophila S2 cells [3]. A distinct set of 5 genes (Dgt2-6) necessary for localizing γ-Tubulin to the spindle but not to the centrosome was identified and subsequently shown to assemble into a stable MT-interacting complex, termed Augmin [62]. Later 3 additional components (Dgt7, Dgt8/Wac, Dgt9) of this complex were identified defining Drosophila Augmin as an 8-subunit complex of approximately 340 kD [63,64]. The components of this complex localize to the interphase centrosome and to the mitotic spindle and are critical for increasing MT density within the spindle. Augmin subunits are not required for the nucleation of MTs in the vicinity of chromosomes, however, they play a role in stabilizing kinetochore-MT bundles thereby contributing to the formation of a robust bipolar spindle [63]. FAM29A, the orthologue of Dgt6, was the first Augmin subunit identified in humans [62,65]. Similar to the Augmin subunits in Drosophila, FAM29A was demonstrated to promote MT nucleation by recruiting γ-TuRCs to the mitotic spindle and to be involved in the maturation of kinetochore fibers. This indicated that Augmin might be a conserved complex and indeed the human counterpart, which also consists of 8 proteins, was later identified independently in 3 different studies [60,63,66]. The components of the complex were named HAUS1-8, where HAUS stands for homologous to Augmin subunits. All HAUS proteins localize to interphase centrosomes and relocate to spindle MTs in mitosis [66]. Consistent with findings in Drosophila, the HAUS complex is critical for increasing MT density within the spindle. Importantly HAUS was shown to interact with the γ-TuRC and to be required for targeting γ-Tubulin to the mitotic spindle. Furthermore, the absence of HAUS leads to reduced tension between sister kinetochores of aligned chromosomes and to the activation of the spindle assembly checkpoint. In Drosophila, chromosome misalignment and mitotic delay only occur when Augmin and centrosomes are absent suggesting that centrosome and spindle-templated MT nucleation are redundant mechanisms. In contrast to these results, depletion of HAUS alone is sufficient to severely impair chromosome segregation in human cells. This indicates that the spindle-templated MT nucleation pathway plays a more important part in cell division in humans than it does in flies. Moreover, in contrast to Drosophila cells, in which centrosomes are not affected in the absence of Augmin, depletion of HAUS leads to MT-dependent centrosome fragmentation in human cells [66]. These additional centrosome defects might explain the stronger mitotic phenotype seen in humans. Although the identification and functional characterization of the Augmin/HAUS complex led to new insights into mitotic spindle assembly, many questions remain to be addressed. While it is indisputable that HAUS recruits the γ-TuRC to the spindle where it nucleates additional MTs in a centrosome-independent manner, the role of HAUS in kinetochore fiber maturation and spindle assembly checkpoint activation remains to be further analyzed. Furthermore it is not yet known how relocation of HAUS to the spindle upon mitotic entry is regulated and if other molecules are involved in binding the HAUS and γ-TuRC complexes to spindle MTs. Currently, it is hypothesized that HAUS8/HICE1 directly binds to MTs and to HAUS6/FAM29A, which in turn binds the γ-TuRC via NEDD1 [63] (Figure 4). However, how these interactions are brought about and whether HAUS is simply responsible for targeting the γ-TuRC to the spindles or also activates MT nucleation or whether other molecules are involved in the regulation of these processes is currently unknown. Moreover it will be interesting to learn whether HAUS components have additional functions at the centrosome, where they localize throughout interphase. HAUS has also been shown to be critical for increasing MT density in the central spindle during anaphase and to be required for the completion of cytokinesis [63]. Future work will hopefully reveal the molecular mechanisms underlying the functions of the Augmin/HAUS complex.

## Unexpected protein associations

To gain insight into the dynamics of this large protein assembly and to elucidate relevant regulatory mechanisms it becomes increasingly critical to explore a proteins function in time and space. Some proteins have initially been classified as so-called core centrosome proteins. These are proteins, e.g. γ-Tubulin or Drosophila Cnn, which remain present on the centrosome throughout a lengthy biochemical centrosome isolation process and whose localization in cells does not require intact MTs. Recent evidence supports the actual dynamic nature of some of the centrosome proteins, for example CNN [67]. Such dynamic processes might reflect the requirement of the centrosome to grow during the process of centrosome maturation at G2/M or to shrink at the M/G1 transition or to regulate the asymmetric acquisition of pericentriolar material (PCM) or other factors between mother and daughter centrioles throughout differentiation [68]. Moreover, protein complexes and proteins (e.g. of the γ-TuSC complex and centrin) previously regarded as *bona fide* centriolar or centrosome proteins are now discovered to have specific functions at the kinetochore [69] or in the nuclear pore complex [70], where they regulate MT plus end capture or mRNA processing. Conversely, proteins (beta-Catenin, Axin) initially described to localize to the nucleus are also found at the centrosome [71,72] and take over functions that relate to centrosome separation or MT nucleation. Unexpected links of centrosome proteins exist to cellular pathways such as DNA damage and DNA replication [73,74] in which for example human CDK5RAP2 is required for DNA damage induced G2 cell cycle arrest. These results provide additional support for the proposition that centrosome proteins might be involved in preventing premature mitotic entry during DNA damage or during incomplete DNA replication. Another unexpected finding was that γ-Tubulin plays an important role in the inactivation of the anaphase-promoting complex/cyclosome (APC/C) in G1 phase of the cell cycle in Aspergillus nidulans. This new regulatory role is apparently independent of MTs since failure of inactivation of the APC/C also occurs in γ-Tubulin mutant strains when MTs are absent. This novel function of γ-Tubulin was revealed through time-lapse microscopy of mitotic regulatory proteins, such as Cdk1 and Cyclin B, which failed to accumulate in mutant strains as a result of permanently active APC/C [75].

## The role of posttranslational modifications of centrosome proteins

There is already good evidence that posttranslational modifications are defining the location, interaction and function of centrosome proteins and protein complexes. Several reports have highlighted the importance of phosphorylation modulating the functions of centrosome proteins. Keck et al. have determined the phosphoproteome of the yeast spindle pole body in different cell cycle stages and demonstrated that Cdk phosphorylation of Spc42, a core component of the yeast centrosome, is critical for centrosome assembly and viability. Furthermore a conserved Cdk site within γ-Tubulin was shown to contribute to proper dynamics of anaphase spindle MTs and mitotic progression [76]. In recent proteomic screens and computational approaches a number of previously unknown substrates of centrosome-associated kinases have been identified [77-79]. Other studies have described phosphorylation-dependent functions of a single centrosome protein. For example, CPAP, the human homologue of Sas-4, which is involved in centriole duplication, has been identified as a Plk2 substrate. Plk2-phosphorylated CPAP localizes to procentrioles and is critical for their elongation [80]. Apart from phosphorylation, other posttranslational modifications, such as SUMOylation and ubiquitylation, play important roles in regulating centrosome biology. Ubiquitylation of the centrosome protein CP110 during G2 phase of the cell cycle and its subsequent degradation is required for centrosome, spindle and genome integrity [81]. Ubiquitin-dependent proteolysis has also been shown to be important for centrosome duplication, procentriole formation and daughter centriole length control [82]. In particular the centriolar protein SAS-6 is required in human cells for procentriole formation regulated through the ubiquitine ligase APC^Cdh1^ that targets SAS-6 for degradation [83] therefore limiting the process of procentriole nucleation. In addition, the E3 ubiquitine ligase complex SCF-FBXW5 that ubiquitylates SAS-6 is negatively regulated by the Polo kinase PLK4 [84]. Therefore PLK4 initiated centrosome duplication also involves phosphorylation of FBXW5. Autophosphorylation of PLK4 itself brings about ubiquitine and proteasome dependent degradation of PLK4, subsequently causing a block of centriolar reduplication by releasing the activity of the SCF-FBXW5 complex. Hence a continuous centriolar replication cycle is governed by a cascade of phosphorylation and proteolytic events [84]. In addition to phosphorylation and ubiquitination, SUMOylation of centrosomal proteins takes place regulating for example the nuclear localization of centrin-2, initially described as a core component of mammalian centrosomes [85]. Ultimately these results emphasize the need for more detailed investigations of posttranslational modifications of centrosome components to better understand the regulatory mechanisms underlying the various aspects of centrosome function and protein localization.

## Future directions and expectations

It is likely that the future direction of centrosome research will continue to be driven by technology developments revealing new gene networks, protein interactions, structural information and functions. Differential protein identification and quantification by MS will permit a more precise definition of the centrosome proteome and particular roles of centrosome proteins throughout the cell cycle and development [7,86]. Current protein isolation techniques so far have provided us mostly with snapshot information on the protein association of the particular bait protein or complex isolated from proliferating cell populations. In the future we will need to employ single-molecule analysis techniques to provide time and space resolved information for elucidating the function and association of proteins at particular positions within the cell and during the cell cycle. Identifying possible disease relevant variants of centrosome proteins via next generation sequencing, high throughput technologies and software improvement for image analysis through automatic microscopy and multiparameter classification [2] will reveal new functional interdependencies and pathway information. Improvements in resolution in light [87] and (cryo-) electron microscopy [88] and EM tomography [89] will reveal more structural details of the centrosome and features of protein complexes allowing us to better understand the function and "mechanics" of the centrosome megacomplex on a molecular level. Beautiful structural studies employing x-ray crystallography, cryo-electron microscopy and rotary metal-shadowing electron microscopy have already provided us with important information on how the centriole and ultimately the centrosome might assemble into a higher order structure. These studies revealed that the highly conserved protein SAS-6 self assembles in vitro into ring-like cartwheel structural elements that resemble the inner core structure of the centriolar cylinder [90,91] and suggest that SAS-6 is critical for establishing the nine fold symmetry of the centriole through its capacity to oligomerize. The combination of the information on the molecular interactions and high-resolution structural information will in the future provide us with a detailed physical map of the centrosome to better understand aspects of its complex assembly, duplication and functions.

## Conclusions

Centrosome biology has experienced a renaissance in recent years with many new PCM and centriolar proteins discovered revealing new and basic principles of centrosome assembly and function for a wide spectrum of organisms. After the protein complement of the centrosome has almost exhaustively been identified we now need to make sense from the large spectrum of different and unexpected components that have been annotated as centrosomal. Much work is still required to reveal the molecular and functional role of the centrosome in cells and tissues throughout differentiation and development of disease.

## Abbreviations

γ-TuRC: γ-Tubulin ring complex; γ-TuSC: γ-Tubulin small complex; GCP: γ-Tubulin complex protein; MT: Microtubule; PCM: Pericentriolar material; APC/C: Anaphase-promoting complex/cyclosome; RNAi: RNA interference, MS: mass spectrometry; PCP: Protein correlation profiling; SILAC: Stable isotope labeling on amino acids; GO: Gene Ontology; EM: Electron microscopy.

## Competing interests

We declare no competing interests.

## Authors’ contributions

K.H. wrote the following sections of the manuscript: Centrosome proteomics, Centrosome functions, Centrosome complexes: γ-Tubulin ring complex and Augmin/HAUS, The role of posttranslational modifications of centrosome proteins. K.H. prepared all figures. B.M.H.L. designed and coordinated the manuscript and wrote the following sections: Abstract, Unexpected protein associations, Future directions and expectations, Conclusions. All authors read and approved the final manuscript.

## Authors’ information

K.H. is a PostDoc who carried out her PhD and subsequent PostDoc work on centrosome proteomics and function of the *Drosophila* centrosome.

B.L. is a group leader focusing on protein complex function in *Drosophila* and in human cells with an emphasis on cell division signaling and disease.
